# Asymmetrically Dominated Choice Problems, the Isolation Hypothesis and Random Incentive Mechanisms

**DOI:** 10.1371/journal.pone.0090742

**Published:** 2014-03-20

**Authors:** James C. Cox, Vjollca Sadiraj, Ulrich Schmidt

**Affiliations:** 1 Andrew Young School of Policy Studies, Georgia State University, Atlanta, Georgia, United States of America; 2 Kiel Institute for the World Economy, Kiel, Germany; 3 Department of Economics, University of Kiel, Kiel, Germany; UC3M, Spain

## Abstract

This paper presents an experimental study of the random incentive mechanisms which are a standard procedure in economic and psychological experiments. Random incentive mechanisms have several advantages but are incentive-compatible only if responses to the single tasks are independent. This is true if either the independence axiom of expected utility theory or the isolation hypothesis of prospect theory holds. We present a simple test of this in the context of choice under risk. In the baseline (one task) treatment we observe risk behavior in a given choice problem. We show that by integrating a second, asymmetrically dominated choice problem in a random incentive mechanism risk behavior can be manipulated systematically. This implies that the isolation hypothesis is violated and the random incentive mechanism does not elicit true preferences in our example.

## Introduction

Under a random incentive mechanism (RIM) subjects usually respond to numerous tasks (e.g. different binary choice questions, bidding for an object in several rounds, etc.) and at the end of the experiment one of the tasks is randomly selected and played out for real. RIM provides incentives for truthfully responding to all tasks while only paying one of them. This reduces expenditures for experimental studies and excludes wealth effects from paying all choices sequentially during the experiment as well as portfolio effects from paying all choices at the end of the experiment. Due to theses appealing features, RIM has been used in many experimental studies in psychology, e.g [1], [2] and economics, e.g. [3]–[5].

However, it was pointed out by Holt [6] for binary choice between lotteries that RIM is not necessarily incentive compatible. If the reduction of compound lottery axiom holds, RIM only provides incentives for truthfully reporting preferences which satisfy the independence axiom. Since there exists abundant evidence that independence is often violated, the argument of Holt challenges RIM seriously. This motivated several experimental studies aiming to test whether RIM does elicit true preferences [7]–[16]. All these studies did not observe serious distortions induced by the use of RIM. A convincing reason for this result is the isolation hypothesis from prospect theory [17] which implies that subjects evaluate each task in a RIM independently of the other tasks.

This note presents a simple experiment which tests isolation and incentive compatibility of RIM in the presence of asymmetrically dominated choice problems. The literature of context-dependent choice has shown that adding asymmetrically dominated alternatives in the set of options can systematically influence choice behavior [18]–[21]. In contrast to these studies, in the present experiment asymmetrically dominated alternatives are not included in the set of options in a given task but asymmetrically dominated choice problems are included in a RIM as additional, independent tasks. Given that isolation holds, choice behavior under RIM in one task should not be influenced by the presence of a different task even if preferences are menu-dependent. Asymmetrically dominated choice problems are understood as follows: Suppose there is a choice between a safe lottery S and a risky lottery R. Then a second choice problem, also consisting of a safe alternative S′ and a risky one R′, *risky-dominates* the first problem if R′ dominates R and S′ is dominated by S. Analogously, a third problem, consisting of a safe alternative S″ and a risky one R″, *safely-dominates* the first one if S″ dominates S and R″ is dominated by R. Our hypothesis is that in the presence of a risky-dominating choice problem alternative R (S) looks less (more) attractive, leading to a higher fraction of S choices. The opposite should hold in the presence of a safely-dominating choice problem.

## Methods

Two experiments were run at the University of Kiel with altogether 581 subjects. Subjects gave written consent to participate in the study. As there was no possibility to lose money in the experiments, approval of an ethics committee was neither required nor obtained. In both experiments subjects were randomly assigned to one of five groups, referred to as Groups 1, 2.1, 2.2, 3.1, and 3.2 in the sequel. For Experiment 1 the stimuli received by the groups (in each case printed on a single sheet of paper) are presented in Table 1.

**Table 1:** Design of Experiment 1.

	Group 1	Group 2	Group 3
	Option A: 4 € with 100%	Option C: 3 € with 100%	Option E: 5 € with 100%
First Choice	Option B:10 € with 50%	Option D:12 € with 50%	Option F: 8 € with 50%
	0 € with 50%	0 € with 50%	0 € with 50%
Second Choice		Option A: 4 € with 100%	Option A: 4 € with 100%
		Option B:10 € with 50%	Option B:10 € with 50%
		0 € with 50%	0 € with 50%

In Group 1 subjects had just to choose between Options A and B. Subjects were told that everybody would receive the payoff of the chosen option in cash directly after the experiment and that the payoff of Option B would be determined by a coin flip. In Groups 2.1 and 3.1 there were two choice problems (presented in the order of Table 1) and a RIM was employed, i.e. there was a first coin flip which determined whether the first or the second choice problem was played out for real and a second coin flip which determined the payoff if one of the risky options (B, D, or F) was chosen. Group 2.2 (3.2) differed from Group 2.1 (3.1) only by the order in which the choices were presented, i.e. the choice between Options A and B was presented first in Groups 2.2 and 3.2. In all groups the left-right positioning of options was randomized. Design of Experiment 2 was identical to that of Experiment 1 with the only exception that the payoff of all safe options (i.e. Options A, C, and E) was increased by one Euro.

The aim of Group 1 is to elicit true preferences of subjects between Options A and B as a design with one choice problem played out for real offers perfect incentives to state true preferences [22]. Also in Groups 2 (3) we elicit preferences between Options A and B which could however be biased as the design here involves additionally a risky-dominating (safely-dominating) choice problem. If the isolation hypothesis holds, the fraction of subjects choosing A should be identical in Groups 1, 2, and 3. If isolation is violated, the additional choice problem in Groups 2 and 3 may influence the choice between A and B. In Group 2 Option A dominates Option C whereas B is dominated by D. Analogous to the evidence of asymmetrically dominated alternatives in the context-dependent choice experiments this could make Option A look more and Option B less attractive, leading to a higher fraction of A choices compared to Group 1. The opposite could be expected for Group 3 as here A is dominated by E whereas B dominates F. Comparing the fraction of B choices in Groups 2 and 3 with those in Group 1 provides a simple and direct test of isolation. In Groups 2 and 3 we observe choices between A and B which are embedded in a different RIM. Therefore, comparing the fraction of B choices in Groups 2 and 3 provides a test on the incentive compatibility of RIM.

## Results

The results of both experiments are presented in Table 2 which states for all groups and both choices the fraction of subjects choosing the risky lottery. First, we can see that in Groups 2.1 and 2.2 indeed by far most subjects choose the risky option D. These subjects may be reluctant to choose B leading to a higher fraction of observed A choices as compared to Group 1. Also in Groups 3.1 and 3.2, most subjects chose as expected the safe option E and for those A could look less attractive.

**Table 2:** Results.

Group	1	2.1	2.2	3.1	3.2
Experiment 1					
N	58	54	54	62	56
% Choice of B	82.8	51.9	59.3	80.6	78.6
% Choice of D (F)		88.9	96.3	12.9	3.6
Experiment 2					
N	61	62	59	57	58
% Choice of B	31.1	29.0	33.9	52.6**	43.1
% Choice of D (F)		87.1	93.2	7.0	5.2

Let us first look at Experiment 1. The differences between choices of B in the single groups are presented in Table 3 along with tests according to the test-statistics of Conlisk [23]. All tests are two-sided and *** (**, *) refers to a significance-level of 1% (5%, 10%). While 82.8% of subjects chose B in Group 1, this fraction reduces to 51.9% and 59.3% in Groups 2.1 and 2.2 respectively. In both cases, the difference is significant. As expected, A turns out to be more attractive in Groups 2 leading to a significant violation of isolation and, therefore, to a failure of isolation. In Group 3 we have expected the opposite effect as in Group 2 but the fraction of B choices is not significantly higher than in Group 1. This may be due to a large fraction of subjects preferring B anyhow and hence due to a ceiling effect. There are also in each case significant differences between the choice of R in Groups 2 and 3. This shows that the choice behavior in a RIM depends strongly on the other tasks involved. All four tests of the RIM (i.e. 2.1 vs. 3.1, 2.1 vs. 3.2, 2.2 vs. 3.1, and 2.2 vs. 3.2) lead to significantly different choice behavior. Therefore, RIM is not incentive-compatible in our experiment.

**Table 3:** Differences in the Choice of B in Experiment 1.

	Group 1	Group 2.1	Group 2.2	Group 3.1	Group 3.2
Group 1	-				
Group 2.1	30.9***	-			
Group 2.2	23.5**	−8.3	-		
Group 3.1	2.2	−28.7***	−21.3**	-	
Group 3.2	−4.2	−26.7***	−19.3**	2.0	-

Ordering effects between Groups 2.1 and 2.2 as well as between Groups 3.1 and 3.2 can be observed which are all in the expected direction, i.e. the choice behavior between A and B should be less affected, if this choice is presented first. However, these effects are insignificant. The relatively small ordering effects can be explained by the fact that in the instructions to Groups 2 and 3 all alternatives were presented prior to the response of subjects.

Table 4 reports the differences of fractions of B choices in the single groups for Experiment 2. Here, in contrast to Experiment 1 we do not observe significant differences between Group 1 and Groups 2 but now the differences between Group 1 and Groups 3 turn out to be significant such that isolation is again violated. Also between Groups 2 and 3 in three out of four cases responses are significantly different which shows that RIM is not incentive compatible.

**Table 4:** Differences in the Choice of B in Experiment 2.

	Group 1	Group 2.1	Group 2.2	Group 3.1	Group 3.2
Group 1	-				
Group 2.1	2.1	-			
Group 2.2	−2.8	−4.9	-		
Group 3.1	−21.5***	−23.6***	−18.7**	-	
Group 3.2	−12.0*	−14.1***	−9.2	9.5	-

## Discussion

This note has shown with a very simple experimental design that integrating asymmetrically dominated alternatives in a random incentive mechanism can manipulate choice behavior systematically. In our study isolation is violated significantly and RIM does not elicit true preferences. We ran eight tests of isolation (fraction of B choices in Group 1 versus the other four groups in both experiments) and observed a significant violation in four cases. This is rather clear-cut and not mixed evidence because isolation cannot be used to justify use of RIM unless it holds generally; holding 50 percent of the time clearly will not do. Additionally, we ran eight tests of the incentive-compatibility of RIM (fraction of B choices in Groups 2 versus Groups 3 in both experiments) and in seven out of these eight tests responses were significantly different. We can conclude that choice behavior in RIM depends substantially on the other tasks involved and asymmetrically dominated alternatives have an impact in the hypothesized direction.

Altogether, the presented results demonstrate that a common methodology in experimental studies may induce distortions. Further research is needed in order to investigate how serious these distortions are in practice.

## References

[1] Birnbaum MH (2008) New Paradoxes of Risky Decision Making. Psychological Review 115: 463–501. 18426300 10.1037/0033-295X.115.2.463

[2] Regenwetter M, Dana J, Davis-Stober CP (2011) Transitivity of Preferences. Psychological Review 118: 42–56. 21244185 10.1037/a0021150

[3] Costa-Gomes MA, Weizsäcker G (2008) State Beliefs and Play in Normal-Form Games. Review of Economic Studies 75: 729–762.

[4] Heinemann F, Nagel R, Ockenfels P (2009) Measuring Strategic Uncertainty in Coordination Games. Review of Economic Studies 76: 181–221.

[5] Offerman T, Sonnemans J, Van de Kuilen G, Wakker P (2009) A Truth Serum for Non-Bayesians: Correcting Proper Scoring Rules for Risk Attitudes. Review of Economic Studies 76: 1461–1489.

[6] Holt CA (1986) Preference Reversals and the Independence Axiom. American Economic Review 76: 508–515.

[7] Camerer CF (1989) An Experimental Test of Several Generalized Utility Theories. Journal of Risk and Uncertainty 2: 61–104.

[8] Starmer C, Sugden R (1991) Does the Random Lottery Incentive System Elicit True Preferences? An Experimental Investigation. American Economics Review 81: 971–978.

[9] Beattie J, Loomes G (1997) The Impact of Incentives upon Risky Choice Experiments. Journal of Risk and Uncertainty 14: 149–62.

[10] Cubitt RP, Starmer C, Sugden R (1998) On the Validity of the Random Lottery Incentive System. Experimental Economics 1: 115–131.

[11] Hey JD, Lee J (2005a) Do Subjects Separate (or Are They Sophisticated)?. Experimental Economics 8: 233–265.

[12] Hey JD, Lee J (2005b) Do Subjects Remember the Past?. Applied Economics 37: 9–18.

[13] Laury SK (2005) Pay One or Pay All: Random Selection of One Choice for Payment. Working Paper 06-13, Andrew Young School of Policy Studies, Georgia State University.

[14] Lee J (2008) The Effect of the Background Risk in a Simple Chance Improving Decision Model. Journal of Risk and Uncertainty 36: 19–41.

[15] Anderson S, Harrison GW, Lau MI, Rutström EE (2007) Valuation Using Multiple Price List Formats. Applied Economics 39: 675–682.

[16] Baltussen G, Post T, van den Assem MJ, Wakker PP (2009) Random Incentive Systems in a Dynamic Choice Experiment. Working Paper, Department of Economics, Erasmus University, Rotterdam, the Netherlands.

[17] Kahneman D, Tversky A (1979) Prospect Theory: An Analysis of Decision under Risk. Econometrica 47: 263–291.

[18] Bhargava I, Kim J, Srivastava R (2000) Explaining Context Effects on Choice Using a Model of Comparative Judgement. Journal of Consumer Psychology 9: 167–177.

[19] Huber J, Payne JW, Puto C (1982) Adding Asymmetrically Dominated Alternatives: Violations of Regularity and the Similarity Hypothesis. Journal of Consumer Research 9: 90–98.

[20] Simonson I, Tversky A (1992) Choice in Context: Tradeoff Contrast and Extremeness Aversion. Journal of Marketing Research 29: 281–295.

[21] Hsee CK, Leclerc F (1998) Will Products Look More Attractive when Evaluated Jointly or When Evaluated Separately?. Journal of Consumer Research 25: 175–186.

[22] Cubitt RP, Starmer C, Sugden R (2001) Discovered Preferences and the Experimental Evidence of Violations of Expected Utility Theory. Journal of Economic Methodology 8: 385–414.

[23] Conlisk J (1989) Three Variants on the Allais Example. American Economic Review 79: 392–407.

